# Supplementary material for: Differences in the Clinical Picture in Women with a Depressive Episode in the Course of Unipolar and Bipolar Disorder
Source: J Clin Med. 2021 Feb 10;10(4):676. doi: 10.3390/jcm10040676 (PMC7916360; doi:10.3390/jcm10040676)
Supplement: Supplementary file 1 [file jcm-10-00676-s001.pdf]

Supplementary Materials:

Table S1. Profile of depressive symptoms based on SCID-I

Symptoms	Participants		p - value of chi-square test
	Unipolar patients	Bipolar patients	
Loose interest	93.33	97.44	0.2184
Weight loss	53.33	69.23	0.0934
Weight gain	16.67	7.69	0.4795
Insomnia	86.67	84.62	0.3621
Hypersomnia	0.00	17.95	**0.0081**
Psychomotor agitation	23.33	20.51	0.7962
Psychomotor retardation	73.33	76.92	0.2673
Lack of energy	96.67	97.44	0.2715
Worthlessness	76.67	82.05	0.2249
Inappropriate guilt	56.67	64.10	0.2170
Diminished ability to think	76.67	92.31	0.0906
Indecisiveness	50.00	56.41	0.2498
Thoughts of own death	70.00	79.49	0.1655
Suicidal ideation	50.00	76.92	**0.0253**
Specific plan	33.33	53.85	**0.0482**
Suicide attempt	36.67	56.41	0.0555

Data presented in column participants report the percentage of depressive symptoms frequency. Chi-square test, significant p-value in bold.

Table S2. Blood test results for patients and controls

Parameters	Unit	Reference range	Bipolar disorder				Unipolar disorder				Control group	
			Pre-treatment		Post-treatment		Pre-treatment		Post-treatment			
			Mean	SD	Mean	SD	Mean	SD	Mean	SD	Mean	SD
CRP	mg/dl	<0.5	**0.69**	1.02	0.43	0.29	**0.79**	1.71	0.41	0.48	0.19	0.33
Urea	mg/dl	17-43	21.53	7.03	21.44	7.76	23.29	10.07	20.19	8.84	27.12	7.92
ALT	U/l	<35	19.12	11.52	22.48	19.67	24.83	25.00	23.75	25.82	17.46	7.04
AST	U/l	<35	20.35	8.16	21.17	8.85	22.09	13.80	23.00	10.63	19.79	6.30
Glucose	mg/dl	74-106	95.23	21.19	86.82	14.64	91.77	16.94	91.29	12.16	92.74	6.30
Triglyceride	mg/dl	35-131.3	**155.70**	90.51	**163.66**	132.87	124.34	55.17	117.85	53.63	90.87	40.48
Total cholesterol	mg/dl	<200	**212.81**	51.77	193.07	43.19	**205.29**	36.06	196.30	43.63	193.58	45.47
HDL	mg/dl	>42	50.33	13.85	49.69	10.82	57.29	16.23	62.63	23.40	68.31	17.20
LDL	mg/dl	<135	131.33	46.57	109.69	37.33	122.71	32.23	115.30	33.91	107.19	41.31
TSH	μIU/ml	0.35-4.94	1.74	1.59	3.02	1.69	1.51	0.95	1.49	0.69	2.25	1.43

Abbreviations: CRP C-reactive protein, ALT alanine transaminase, AST aspartate transaminase, HDL high-density lipoprotein, LDL low-density lipoprotein, TSH thyroid-stimulating hormone. Values greater than the reference range are in bold.

Table S3. Statistical analyses for blood test results for patients and controls

Parameters	**BP** p-value of Wilcoxon test	**UP** p-value of Wilcoxon test	**Pre-treatment** p-and (z)-value of post-hoc Dunn test BP vs UP	BP vs CG	UP vs CG	p-value of Kruskal-Wallis test	**Post-treatment** p- and (z)-value of post-hoc Dunn test BP vs UP	BP vs CG	UP vs CG	p-value of Kruskal-Wallis test
CRP	0.2772	0.5408	1.0000 (0.5137)	**0.0000 (6.9239)**	**0.0000 (5.8694)**	**0.0000**	1.0000 (0.8603)	**0.0000 (5.8963)**	**0.0002 (3.9685)**	**0.0000**
Urea	0.6148	0.2489	0.6120 (1.2703)	**0.0030 (3.2860)**	0.2550 (1.7224)	**0.0038**	1.0000 (0.0818)	**0.0071 (3.0379)**	**0.0345 (2.5270)**	**0.0017**
ALT	0.4195	0.0640	1.0000 (0.4782)	1.0000 (0.2641)	1.0000 (0.2717)	0.8915	1.0000 (0.2763)	1.0000 (0.6342)	1.0000 (0.1974)	0.8162
AST	0.9785	0.1424	1.0000 (0.1342)	1.0000 (0.8446)	1.0000 (0.6780)	0.6734	1.0000 (0.8383)	1.0000 (0.5121)	0.5487 (1.3319)	0.4013
Glucose	0.1059	0.9702	0.9474 (0.8878)	1.0000 (0.4012)	0.1836 (1.2157)	0.4556	1.0000 (0.6702)	**0.0389 (2.5081)**	0.4855 (1.4873)	**0.0335**
Triglyceride	0.9397	0.9357	0.8060 (1.1061)	**0.0000 (4.5945)**	**0.0059 (3.0972)**	**0.0000**	0.2443 (1.7425)	**0.0000 (4.4195)**	0.0705 (2.2653)	**0.0000**
Total cholesterol	0.1945	0.9393	1.0000 (0.1883)	0.0689 (2.2739)	0.1627 (1.9249)	**0.0378**	1.0000 (0.5145)	1.0000 (0.1856)	1.0000 (0.7846)	0.7342
HDL	0.3414	0.1840	0.4287 (1.4651)	**0.0000 (5.1794)**	**0.0034 (3.2534)**	**0.0000**	**0.0425 (2.4531)**	**0.0000 (5.2413)**	0.0767 (2.2329)	**0.0000**
LDL	0.0757	0.2692	1.0000 (0.2718)	**0.0071 (3.0387)**	**0.0306 (2.5693)**	**0.0029**	1.0000 (0.6863)	1.0000 (0.5844)	0.5074 (1.3750)	0.3817
TSH	**0.0268**	0.2076	1.0000 (0.1671)	**0.0150 (2.8067)**	**0.0139 (2.8320)**	**0.0029**	**0.0499 (2.3945)**	0.3122 (1.6254)	0.3736 (1.5360)	0.0533

Abbreviations: CRP C-reactive protein, ALT alanine transaminase, AST aspartate transaminase, HDL high-density lipoprotein, LDL low-density lipoprotein, TSH thyroid-stimulating hormone. P- and z-value of post hoc test are given in the same row, z-value is in brackets. Significant p- and z-value ($p<0.05$) in bold.

Figure S1. Frequency of chronotypes in patients and controls.

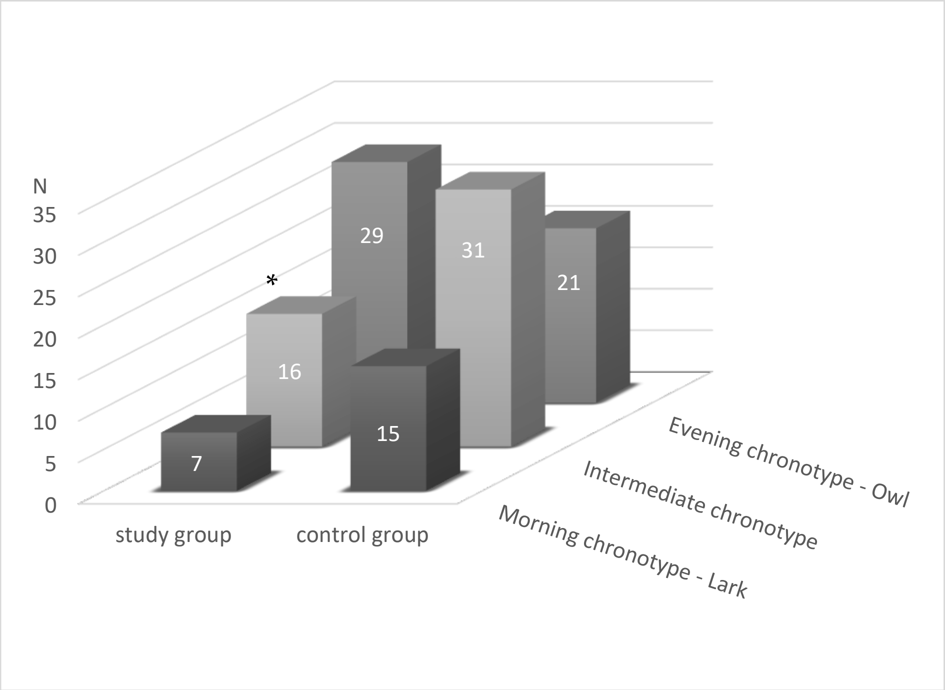

* statistically significant difference between study and control group, Mann-Whitney U test, p=0.0194

Table S4. The Barrat Impulsiveness scale scores of post-treatment psychiatric patients

Disease type/Scale	Bipolar Disorder		Unipolar Disorder		p-value
	Mean	SD	Mean	SD	
Total score	65.04	4.58	59.3	7.7	**0.0152**
**Second-order factor**					
Attentional Impulsiveness	16.65	2.17	16.1	3.3	0.5471
Motor Impulsiveness	21.73	2.44	18.8	3.5	**0.0042**
Nonplaning Impulsiveness	26.65	3.40	24.4	4.1	0.0841
**First-order factors**					
Attention	10.46	1.69	10.2	2.2	0.6761
Motor impulsiveness	14.31	2.28	12.4	3.2	**0.0206**
Self-control	15.81	2.59	14.6	2.5	0.1454
Cognitive complexity	10.85	1.34	9.8	2.2	0.1124
Perseverance	7.42	1.22	6.4	1.8	0.0547
Cognitive instability	6.19	1.28	5.8	1.8	0.5748

Mann-Whitney U test, significant p-value (p<0.05) in bold

Table S5. BLEQ differences between bipolar disorder group, major depression disorder group and control group (%)

Event	Participants			p-value		
	BP	UP	CG	BP vs UP	BP vs CG	UP vs CG
1. Serious illness. injury or an assault	35.48	45.45	14.93	0.8273	**0.0252**	**0.0098**
2. Serious illness. injury or an assault of close relative	12.90	22.73	17.91	0.7389	0.6625	0.6744
3. Death of a spouse or first-degree relative	0.00	18.18	0.00	**0.0455**	1.0000	**0.0005**
4. Death of a close family friend or second-degree relatives	12.90	27.27	10.45	0.5271	0.64511	0.0782
5. Separation due to marital difficulties or break up of a steady relationship	25.81	22.73	1.49	0.4054	**0.0001**	**0.0009**
6. Serious problem with a close friend, neighbour or relatives	38.71	18.18	16.42	**0.0455**	**0.0203**	0.8815
7. Job loss	9.68	0.00	1.49	0.0833	**0.0495**	0.5637
8. Seeking work without success for more than 1 month	16.13	18.18	5.97	0.7389	0.0943	0.1025
9. Major financial crisis	12.90	9.09	4.48	0.4142	0.1171	0.4386
10. Problems with the police involving a court appearance	0.00	4.55	2.99	0.3173	0.3545	0.7389
11. Valued item lost or stolen	6.45	0.00	8.96	0.1573	0.7576	0.1573
12. The birth of a child	6.45	0.00	2.99	0.1573	0.3827	0.4142

BP - bipolar disorder, UP - unipolar disorder, CG - control group. Data presented in column participants report the percentage of frequency events. Significant p-value in bold, chi-square test

Table S6. Comparison of the COPE results in post-treatment patients and control group

Strategy	patients		literature controls*		p-value	Bipolar disorser		Unipolar disorder		p - value
	Mean	SD	Mean	SD		Mean	SD	Mean	SD	
Active coping	10.68	2.20	11.36	1.95	0.0721	10.44	2.61	11.00	1.54	0.3071
Planning	10.61	2.08	11.47	2.35	0.0569	10.38	2.25	10.92	1.88	0.6931
Seeking Instrumental Social Support	10.64	3.12	11.64	2.43	**0.0351**	10.88	2.85	11.08	2.61	0.8164
Seeking Emotional Social Support	10.96	2.71	11.37	2.45	0.3872	10.88	2.87	10.33	3.55	0.5934
Suppression of Competing Activities	10.43	2.66	10.63	1.97	0.6037	10.31	2.94	10.58	2.35	0.9815
Turning to Religion	8.61	3.79	9.17	3.65	0.4267	8.31	3.46	9.00	4.33	0.7452
Positive Reinterpretation and Growth	9.43	2.44	11.1	1.96	**<0.0001**	9.06	2.38	9.92	2.54	0.4300
Restraint Coping	9.86	2.35	10.45	1.73	0.0816	9.56	2.31	10.25	2.45	0.3071
Acceptance	10.14	3.03	9.71	2.3	0.3385	10.06	3.23	10.25	2.86	0.8892
Focus on and Venting Emotions	11.68	2.18	11.13	2.1	0.1750	11.75	2.27	11.58	2.15	1.0000
Denial	7.25	1.82	7.11	2.12	0.7307	7.13	2.09	7.42	1.44	0.4576
Mental Disengagement	9.18	1.72	8.75	2.12	0.2897	9.13	1.67	9.25	1.86	0.8709
Behavioral Disengagement	8.71	2.99	7.07	2.17	**0.0001**	8.88	2.92	8.50	3.21	0.5462
Alcohol/Drug Use	5.25	2.49	5.11	2.37	0.7596	5.94	3.00	4.33	1.15	0.1858
Humor	5.93	2.26	6.31	2.4	0.4104	5.63	2.09	6.33	2.50	0.3904

Comparison between patients and controls were tested by two-sided comparison of two means test. The p-value in bold are statistically significant.* Values of controls are literature data according to Juczyński and Ogińska-Bulik, [38]. Group disorders were tested by U-Mann Whitney test.

Table S7. Antidepressant Side-Effect Checklist results for a bipolar and unipolar group in depression and euthymic state (%)

Variables	Percentages of incidence scores				p-value of comparition			
	Pre-treatment		Post-treatment		between disorder		between Pre- and Post-treatment	
	Bipolar	Unipolar	Bipolar	Unipolar	Pre-treatment	Post-treatment	Bipolar	Unipolar
1 Dry mouth	40.74	0.00	15.15	0.00	**0.0225**	0.3373	**0.0431**	1.0000
2 Drowsiness	22.22	16.67	3.03	0.00	0.7293	0.8568	**0.0431**	0.1797
3 Insomnia	55.56	44.44	9.09	4.55	0.6598	0.7834	**0.0033**	**0.0180**
4 Blured vision	22.22	0.00	6.06	0.00	0.2152	0.7160	0.1422	1.0000
5 Headache	33.33	22.22	9.09	9.09	0.5667	0.9931	**0.0277**	0.1088
6 Constipation	40.74	22.22	15.15	4.55	0.2670	0.4979	**0.0431**	0.1088
7 Diarrhoea	3.70	0.00	6.06	0.00	0.8439	0.7118	0.7395	1.0000
8 Increased appetite	11.11	22.22	30.30	27.27	0.5392	0.9247	**0.0277**	0.7794
9 Decreased appetite	44.44	50.00	9.09	0.00	0.7633	0.5766	**0.0269**	**0.0077**
10 Nusea or Vomiting	0.00	0.00	6.06	4.55	1.0000	0.9434	0.1797	0.8107
11 Urination problems	11.11	5.56	3.03	0.00	0.7633	0.8568	0.1088	0.7753
12 Sexual problems	33.33	22.22	15.15	9.09	0.5392	0.7118	0.1422	0.1797
13 Palpitations	48.15	22.22	24.24	13.67	0.1476	0.5139	**0.0277**	0.2249
14 Orthostatic dizziness	33.33	16.67	6.06	0.00	0.4108	0.7118	**0.0382**	0.1088
15 Vertigo	22.22	5.56	3.03	0.00	0.3541	0.8568	**0.0431**	0.7753
16 Sweating	7.41	11.11	6.06	4.55	0.8439	0.9315	0.8843	0.5930
17 Increased temperature	7.41	0.00	0.00	0.00	0.6761	0.9931	0.1797	1.0000
18 Tremor	22.22	16.67	30.30	4.55	0.7633	0.1101	0.4631	0.1797
19 Disorientation	11.11	0.00	0.00	0.00	0.5392	0.9931	0.1088	1.0000
20 Yawning	37.04	22.22	15.15	18.18	0.4108	0.8568	0.0910	0.7998
21 Weight gain	25.93	22.22	24.24	18.18	0.8021	0.7118	0.7353	0.6858

Statistical important differences ($p<0.05$) are in bold. In comparison between depression and euthymic state Wilcoxon test was applied, whereas differences between bipolar and unipolar groups were tested by U-Mann Whitney test.
